# Factors associated with benign multiple sclerosis in the New York State MS Consortium (NYSMSC)

**DOI:** 10.1186/s12883-016-0623-2

**Published:** 2016-07-15

**Authors:** Robert Zivadinov, Diane L. Cookfair, Lauren Krupp, Aaron E. Miller, Neil Lava, Patricia K. Coyle, Andrew D. Goodman, Burk Jubelt, Michael Lenihan, Joseph Herbert, Malcolm Gottesman, David H. Snyder, Brian R. Apatoff, Barbara E. Teter, Allan B. Perel, Frederick Munschauer, Bianca Weinstock-Guttman

**Affiliations:** Buffalo Neuroimaging Analysis Center, Department of Neurology, School of Medicine and Biomedical Sciences, University at Buffalo, Buffalo, NY USA; Department of Neurology, School of Medicine and Biomedical Sciences, University at Buffalo, 100 High Street, Buffalo, NY 14203 USA; Department of Neurology, Stony Brook University Medical Center, Stony Brook, NY USA; The Corinne Goldsmith Dickinson Center of Multiple Sclerosis, Mount Sinai School of Medicine, New York, NY USA; Department of Neurology, Albany Medical School, Multiple Sclerosis Center, Albany, NY USA; Department of Neurology, University of Rochester Medical Center, Rochester, NY USA; Department of Neurology, SUNY Upstate Medical University, Syracuse, NY USA; Adirondack Neurology Associates, Glens Falls, NY USA; Department of Neurology, NYU School of Medicine, New York, NY USA; Department of Neuroscience, Winthrop University Hospital, Mineola, NY USA; Department of Neurology, Weill Medical College of Cornell University, New York, NY USA; Department of Neurology, Albert Einstein College of Medicine, New York, NY USA; Multiple Sclerosis Center, New York, NY USA; Alpha Neurology, Staten Island, NY USA

**Keywords:** Benign multiple sclerosis, Disease course, Disease-modifying treatment, DMT, Multiple sclerosis

## Abstract

**Background:**

This retrospective analysis explored prognostic factors associated with a benign multiple sclerosis (BMS) disease course at baseline and over the 4-year follow-up.

**Methods:**

Patients from the centralized New York State Multiple Sclerosis Consortium registry were classified as having BMS according to 3 different criteria centered on disease duration and disability. Additional analyses explored prognostic factors associated with BMS using the most conservative disability criteria (Expanded Disability Status Scale ≤2 and disease duration ≥10 years).

**Results:**

Among 6258 patients who fulfilled eligibility criteria, 19.8 % to 33.3 % were characterized as having BMS, at baseline depending on classification criteria used. Positive prognostic factors for BMS at baseline included female sex (*p <* 0.0001) and younger age at onset (*p <* 0.0001); negative prognostic factors included progressive-onset type of MS and African-American race. Of the 1237 BMS patients (per most conservative criteria), 742 were followed for a median of 4 years to explore effect of disease-modifying treatment (DMT) on benign status. DMT (*p =* 0.009) and longer disease duration (*p =* 0.007) were the only significant positive predictors of maintaining BMS at follow-up. The protective effect was stronger for patients taking DMT at both enrollment and follow-up (OR = 0.71; *p* = 0.006).

**Conclusions:**

There is a need for development of more reliable prognostic indicators of BMS. Use of DMT was significantly associated with maintaining a benign disease state.

**Electronic supplementary material:**

The online version of this article (doi:10.1186/s12883-016-0623-2) contains supplementary material, which is available to authorized users.

## Background

Multiple sclerosis (MS) is an immune-mediated neurodegenerative disorder of the central nervous system with a lifetime risk of 1 in 400 in Caucasians in industrialized society [1]. The disease course is highly heterogeneous and includes a subgroup of patients with benign MS (BMS) who demonstrate little disease progression and minimal disability, even decades after clinical onset [2–5]. However, there is no universally agreed upon definition of BMS, as multiple patient profiles are associated with this disease course [2].

In 1996, an international survey conducted by the National Multiple Sclerosis Society (NMSS) defined BMS as a disease in which patients remain fully functional across neurologic systems 15 years after disease onset [6, 7]. Unfortunately, the term “fully functional” was not clearly defined, resulting in considerable variations in the estimated frequency of BMS [8–10].

Benign MS is a retrospective diagnosis that can only be made 10 years or more after disease onset. Despite consensus recommendations for immediate treatment following diagnosis, there is still a difference of opinion among physicians regarding this issue because of the paucity of efficacy data with disease modifying therapies (DMTs) in patients with BMS [11–15].

The New York State MS Consortium (NYSMSC) has assembled a centralized registry of demographic and clinical information from more than 7000 patients with clinically definite MS from across New York State (NYS), providing a robust dataset to study the frequency and characteristics of BMS. [16, 17] This study aimed to (a) retrospectively estimate the prevalence of BMS based on the NYSMSC registry using the 3 most commonly used BMS criteria [2, 3, 18]; (b) evaluate prognostic factors associated with BMS; (c) investigate the course of BMS prospectively over time [2, 8, 19]; and (d) explore whether DMT use correlated with maintaining a benign status in patients classified as having BMS at time of study enrollment.

## Methods

### Study details

This was a retrospective cohort study characterized by demographic, clinical, and treatment information available from the NYSMSC centralized registry. The NYSMSC is a regional affinity group of 18 MS centers throughout NYS established to promote MS research and enhance patient care [16, 20]. This study consisted of 2 parts: a cross-sectional comparison of patients at the time of enrollment in the registry (i.e. baseline evaluation) and the assessment of patients with follow-up data. Patient enrollment in the NYSMSC registry began on February 14, 1996. This date was also the start of study enrollment. All patients with a clinically definite MS diagnosis (CDMS) according to the Poser criteria [21] enrolled in the NYMSC registry from the start of the registry until December 31, 2002, were included in the study cohort. While this later date represented the final enrollment date for inclusion in the cohort (i.e. December 31, 2002), a follow-up cut-off date of October 1, 2004, was imposed for patient follow-up data. This follow-up cut-off date was chosen to provide similar diagnosis criteria (CDMS), at least 1 year of follow-up for the most recently enrolled patients, and because it represented approximately 10 years since the first DMT approved for treatment of MS became available. A complete baseline evaluation at NYSMSC enrollment, including completion of the demographic and clinical sections of the NYSMSC standardized data collection instrument, was also required for study inclusion.

The standardized data collection instrument consists of 2 sections: Section 1 contains demographic data and self-report assessments that are completed by patients during their regularly scheduled neurologic appointment; Section 2, completed by the examining neurologist and/or nurse practitioner certified in Expanded Disability Status Scale (EDSS) performance, includes signs and symptoms at onset, relapse history, MS type, EDSS score, and use of DMTs. All clinical, demographic, and treatment data from enrollment and yearly (±6 months) follow-up visits from all participating NYSMSC sites are maintained at a centralized data management center (UDSMR) [16, 17].

Patients were defined as receiving DMT, if they received any of the following Food and Drug Administration (FDA)-approved treatments and/or investigational DMTs: FDA-approved DMTs—interferon beta-1a/b (IFN-β1a/b; Avonex®, Rebif®, Betaseron®), glatiramer acetate (Copaxone®), mitoxantrone (Novantrone®), and natalizumab (Tysabri®); investigational DMTs (taken by ~1 % of patients)—cyclophosphamide, intravenous immunoglobulin, oral myelin, linomide, and azathioprine. All patients receiving treatments that were not DMTs (e.g. symptomatic medications) were included in the not-treated group for the various time points unless they also received a DMT.

The study was approved by the relevant Institutional Review Boards (IRBs) of the participating centers and written informed consent was obtained by all study participants prior to enrollment in the NYSMSC registry. The IRB renewals were obtained annually on an ongoing basis throughout the study. The permission was obtained for using the NYSMSC database by the NYSMSC Scientific Committee, which approved the study protocol.

### Patient classification

Patients were retrospectively classified as having BMS or non-BMS at baseline (i.e. NYSMSC enrollment) according to the 3 most commonly used benign criteria: EDSS ≤2 and disease duration (DD) ≥10 years (Criterion I), EDSS ≤3 and DD ≥15 years (Criterion II), and EDSS ≤3 and DD ≥10 years (Criterion III) (Table 1) [2, 3, 18]. Disease duration was defined as time from first symptom onset. Prevalence of BMS at enrollment according to these 3 classifications was evaluated. Additional baseline and follow-up analyses were carried out for the first benign classification criteria (EDSS ≤2 and DD ≥10 years) because this classification was the most conservative with respect to EDSS (referred to herein as Criterion I). Within the context of this classification, patients were placed into 1 of 2 categories: BMS (i.e. EDSS ≤2 and DD <10 years) and non-BMS (i.e. EDSS ≤2 and DD <10; years or EDSS >2 regardless of DD). For this latter category, the worsening to EDSS >2 could have occurred at any time during the disease course.

**Table 1:** Baseline demographic and clinical characteristics of patients with benign MS based on 3 classification criteria

Variable	EDSS ≤2, DD ≥10^a^ (Criterion I)	EDSS ≤3, DD ≥15 (Criterion II)	EDSS ≤3, DD ≥10 (Criterion III)
Patients, n (%)^b^	1237 (19.8)	1253 (20.0)	2081 (33.3)
Female, %	79.6	78.5	77.4
Race, % Caucasian/Hispanic-American African-American Other	94.6 3.8 1.6	95.3 3.6 1.1	94.2 4.3 1.3
Family history,%	19.5	20.6	20.2
Age at onset, y, mean (SD)	30.2 (8.5)	29.3 (8.6)	30.8 (8.9)
Age at baseline, y, mean (SD)	47.8 (9)	51.9 (9)	49.3 (9.5)
EDSS, mean (SD)	1.4 (0.6)	2.1 (0.8)	2.0 (0.8)
EDSS, median (range)	1.5 (0–2.0)	2.0 (0–3.0)	2.0 (0–3.0)
Disease duration, y, mean (SD)	17 (6.7)	22 (6.9)	17.9 (7.3)
Disease course, % Relapsing-remitting Secondary-progressive Progressive-relapsing Primary-progressive	95.6 2.7 0.6 1.1	86.4 9.3 1.8 2.5	88.4 7.5 1.5 2.6
On DMT at enrollment, n (%)^c^	505 (40.8)	481 (38.4)	848 (40.7)
Type of DMT, n (%) Interferon beta-1a IM Interferon beta-1b SC Interferon beta-1a SC Glatiramer acetate Methotrexate Myelin (oral) Mitoxantrone Azathioprine Cyclophosphamide Immunoglobulin (intravenous) Linomide	314 (62.2) 98 (19.4) 1 (0.2) 64 (12.7) 8 (1.6) 13 (2.6) 0 3 (0.6) 1 (0.2) 2 (0.4) 1 (0.2)	300 (62.4) 90 (18.7) 1 (0.2) 59 (12.3) 12 (2.5) 11 (2.3) 0 5 (1.0) 1 (0.2) 1 (0.2) 1 (0.2)	515 (60.7) 164 (19.3) 3 (0.4) 107 (12.6) 19 (2.2) 24 (2.8) 0 7 (0.8) 3 (0.4) 4 (0.5) 2 (0.2)

*DD* disease duration (years from symptom onset to enrollment)

*Abbreviations: DMT* disease-modifying therapy, *EDSS* Expanded Disability Status Scale, *MS* multiple sclerosis

^a^Most conservative definition

^b^Out of *n =* 6258 total cohort patients

^c^Were being treated with disease modifying therapy (DMT) at time of enrollment in the NYSMSC registry

**Table 2:** Baseline demographic and clinical characteristics of MS patients with benign MS according to classification Criterion I^a^ (EDSS ≤2 and DD ≥10 years) vs. non-benign MS patients

Variable	BMS (EDSS ≤2, DD ≥10)^a^	Non-BMS	*p*-value*
All Patients, n (%)^b^	1237 (19.8)	5021 (80.2)	<0.0001
Female, n (%)	985 (79.6)	3590 (71.4)	<0.0001
Race, n, (%) Caucasian/Hispanic-American African-American Other	1170 (94.6) 47 (3.8) 20 (1.6)	4617 (91.9) 329 (6.6) 75 (1.5)	<0.0001
Family history, n, (%)	241 (19.5)	976 (19.4)	NS
Age at onset, y, mean (SD)	30.2 (8.5)	32.2 (9.7)	<0.0001
Age at baseline, y, mean (SD)	47.8 (9.0)	51.8 (10.5)	<0.0001
EDSS, mean (SD)	1.4 (0.6)	4.4 (1.6)	<0.0001
EDSS, median (range)	1.5 (0–2.0)	5.0 (0–9.5)	<0.0001
Disease duration, y, mean (SD)^c^	17.0 (6.7)	18.3 (7.5)	<0.0001
Disease course,(n)%^d^ Relapsing-remitting Secondary-progressive Progressive-relapsing Primary-progressive	1184 (95.6) 33 (2.7) 7 (0.6) 13 (1.1)	2692 (53.6) 1622 (32.3) 219 (4.4) 488 (9.7)	<0.0001
DMT at enrollment, n (%)^e^	505 (40.8)	2182 (81.2)	*p <* 0.0001
Type of DMT, n (%) Interferon beta-1a IM Interferon beta-1b SC Interferon beta-1a SC Glatiramer acetate Methotrexate Myelin (oral) Mitoxantrone Azathioprine Cyclophosphamide Immunoglobulin (intravenous) Linomide T-cell vaccine	314 (62.2) 98 (19.4) 1 (0.2) 64 (12.7) 8 (1.6) 13 (2.6) 0 (0.0) 3 (0.6) 1 (0.2) 2 (0.4) 1 (0.2) 0 (0.0)	1063 (48.7) 466 (21.4) 16 (0.7) 306 (14.0) 206 (9.4) 19 (0.9) 20 (0.9) 39 (1.8) 23 (1.1) 4 (0.2) 19 (0.8) 1 (<0.1)	

*Abbreviations: MS* multiple sclerosis, *BMS* benign MS, *DMT* disease-modifying therapy, *EDSS* Expanded Disability Status Scale, *IM* intramuscular, *NS* not significant, *SC* subcutaneous, *SD* standard deviation

*Between-group comparisons were performed using chi-square, t-tests, or Mann–Whitney U tests as appropriate

^a^Most conservative classification criteria definition

^b^Denominators: Benign MS (*n =* 1237); Other MS (*n =* 5021)

^c^DD, disease duration (years from symptom onset to enrollment)

^d^At time of baseline enrollment, only 53 progressive patients (SP, PR, and PP) out of the entire cohort of 6,258 (<1 %) met classification Criterion Ia (EDSS ≤2 and DD ≥10 years) for benign MS. Removing these patients from the analysis made no difference in statistical significance of results, nor did considering them non-benign due to their status as progressive patients

^e^DMT at enrollment, *n =* 2687 (42.9 %) of total cohort (*n =* 6258) *(benign MS on DMT, n = 505; other MS on DMT n = 2182)*

**Table 3:** DMT use as a predictor of continued benign status at follow-up in those who were benign at enrollment (*n =* 742)^a,b,c,d^

(A) DMT use Ever(*n =* 633)/Never (*n =* 109)^e,f^		
	HR (95 % CI)	*p* value
DMT use at enrollment and/or follow-up Disease duration (years) at enrollment	0.60 (0.42-0.85) 0.97 (0.97–0.99)	0.009 0.007
(B) DMT use 4-category Cox model^e,f^		
	HR (95 % CI)	*p* value
DMT use at enrollment only (*n =* 83) DMT use at follow-up only (*n =* 258) DMT use at both enrollment and follow-up (*n =* 292) Disease duration (years) at enrollment	0.97 (0.74–2.38) 0.80 (0.64–0.99) 0.71 (0.57–0.88) 0.98 (0.97–0.99)	NS 0.037 0.006 0.006

*CI* confidence interval, *DMT* disease-modifying therapy, *HR* hazard ratio *MS* multiple sclerosis

^a^Cox regression analyses

^b^The following variables were entered into the full Cox models comparing late benign with benign patients: sex, disease course, race, treatment status, age at onset and disease duration

^c^Continuous variables: age at onset (years), disease duration (years since symptom onset)

^d^Categorical variables: sex: female compared with male; disease course: secondary-progressive, progressive-relapsing, primary-progressive, compared with relapsing-remitting MS; race: African-American, Other, compared with Caucasian; treatment categories: See footnote f, below

^e^Forty-six of 788 Criteria I baseline benign follow-up patients were excluded from Cox regression analyses, leaving a total of *N =* 742 patients; *N =* 42 patients who were not on treatment at enrollment or follow-up but had a history of IFN-β1a use prior to its approval in 1996 as a result of participation in the IFN-β1a phase 3 trial, and *N =* 4 patients who were missing DMT data

Never Users: *n =* 109 patients had no history of DMT use at enrollment and did not use DMT at any time during the study. This group served as the reference category for DMT use in the Ever/Never and 4-category Cox models. DMTs used included interferon beta-1a/b (Avonex®, Rebif®, Betaseron®), glatiramer acetate (Copaxone®), azathioprine, cyclophosphamide, intravenous immunoglobulin, methotrexate, linomide, myelin therapy; mitoxantrone, and natalizumab (1 patient on clinical trial)

^f^There was no difference in likelihood of remaining benign vs converting to late non-benign during follow-up according to sex, disease course, race, or age at onset after adjusting for significant predictors included in the final model

### Statistical methods

All statistical analyses were performed using Statistical Package for Social Sciences (SPSS Incorporated, Chicago, IL, version 22.0). The association between clinical and demographic variables for Criterion I benign, vs. non-BMS patients was first assessed using chi-square, *t*-test or Mann–Whitney *u* test, as appropriate.

Odds ratios and 95 % confidence intervals for predictors of benign vs. non-BMS status adjusted for other predictors at baseline were calculated using binary logistic regression (LR). For these comparisons, non-BMS patients were limited to those with ≥10 years DD so as to make the two groups similar with regard to minimum disease duration. Cox regression models were used to explore the effect of DMT use from enrollment through follow-up on benign status over time while simultaneously adjusting for significant baseline predictors of benign status in a subset of Criterion I benign patients with follow-up data (*n =* 742). Hazard ratios (HRs) and 95 % confidence intervals were calculated from final Cox models. Backward stepwise modeling was used for both LR and Cox regression, with entry criteria set at *p <* 1.00 and removal criteria at *p <* 0.05. Additional details regarding regression models are provided in the footnotes of Table 3.

**Fig. 1:**
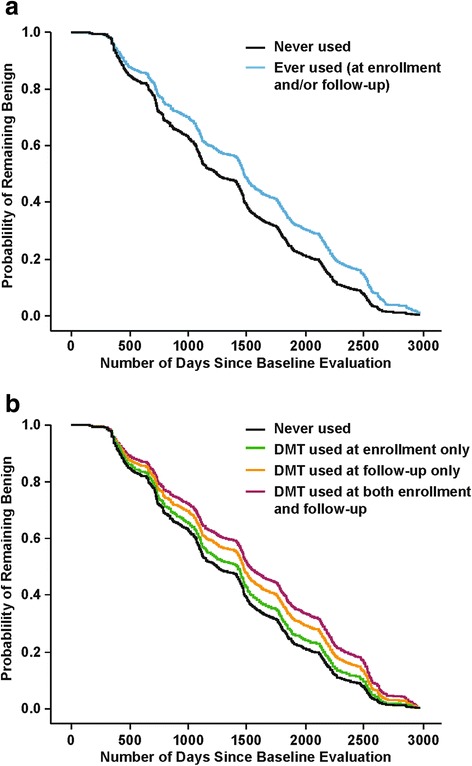
Cox regression survival curves. Probability of remaining benign for *n =* 742 Criterion I benign MS patients for (**a**) 2-category definition of DMT use: Ever (*n =* 633) vs Never (*n =* 109; *p* = 0.009) and (**b**) 4-category definition of DMT use: Never Used (*n =* 109, comparison group); at enrollment only (*n =* 83, not significant); at follow-up only (*n =* 258, *p* = 0.038); at both enrollment and follow-up (*n =* 292, *p* = 0.006). *Abbreviations:* DMT, disease-modifying therapy. Data from New York State Multiple Sclerosis Consortium

All models were initially run with a full set of variables identified as predictors during univariate analyses, with one exception. Because age-related variables were highly correlated, some were eliminated from the model to ensure that final fitted models met the underlying assumptions of multicollinearity and Homer-Lemshow goodness-of-fit criteria (LR). Detailed information regarding variables included in the models are provided in the footnotes for Table 3 and Fig. 1.

Data regarding DMTs taken by individual Criterion I patients included type of DMT taken at time of enrollment and follow-up. Data regarding specific duration of DMT use were not available. Therefore, 2 analyses were conducted. In the first, DMT use was classified as Ever (*n =* 633) or Never (*n =* 109) used as of the end of follow-up. For the second analysis, 4 categories were established using all DMT data as of follow-up: (1) Never used DMT (*n =* 109), (2) Was using DMT at time of enrollment but not at follow-up (*n =* 83), (3) Used DMT at time of follow-up but not on DMT at enrollment (*n =* 258), and (4) Was on DMT at enrollment and also at follow-up (*n =* 292). For this second analysis, the Never used DMT group served as the reference category for the other 3 groups. Forty-two benign patients from the Buffalo, NY, site who were not on treatment at enrollment or follow-up had a history of IFN-β1a use before its approval in 1996 as a result of participation in the IFN-β1a phase 3 trial. These patients (*n =* 42) were excluded from the Cox model analyses exploring the effect of DMTs on benign status. Four additional patients were excluded from Cox model analyses because of missing data on DMT use at enrollment.

## Results

### Demographic and clinical characteristics at baseline

Of the 7158 patients enrolled in the NYSMSC registry, 6258 patients fulfilled the inclusion criteria and were included in the study (Additional file 1: Figure S1). The majority (61.9 %) of patients in the study cohort had relapsing-remitting (RRMS), while approximately one quarter of patients (26.5 %) had secondary-progressive MS (SPMS). Only 8.0 % of patients had primary-progressive MS (PPMS), and <4 % had progressive-relapsing (PR) MS (Additional file 1: Figure S1). Disease course was determined per NMSS recommended criteria available at the time of study enrollment [6].

At baseline, 19.8 % (*n =* 1237) of patients in the total study cohort were classified as having BMS according to the most conservative classification criteria (Criterion I; Table 1). Similarly, 20 % (*n =* 1253) of patients were classified as having BMS using Criterion II. In contrast, when using the least conservative classification (Criterion III), 33.3 % (*n =* 2081) of patients were classified as having BMS (Table 1).

The descriptive data in Table 1 suggest that some demographic and clinical characteristics differed according to criteria, especially for Criterion I vs Criterion II. Certain differences were expected based on criteria definitions (e.g. lower mean EDSS for benign patients in the Criterion I group vs those in Criterion II and III groups, and shorter mean disease durations for BMS in the Criterion I [17 years] and III groups [17.9 years] compared with those in the Criterion II group [22 years]). Disease course also varied for patients defined as having BMS based on Criterion I vs Criterion II and III (Table 1). The percentage of patients with RRMS was higher among Criterion I BMS patients (95.6 %) than among Criterion II (86.4 %) and Criterion III (88.4 %) BMS patients.

In the total cohort of MS patients (*n* = 6258), use of the most conservative classification criteria (Criterion I) resulted in 19.8 % of patients classified as benign at baseline. 80.2 % of patients were classified as having non-BMS (Table 2). In univariate analyses comparing Criterion I BMS patients with non-BMS patients at baseline, significant differences between patients with BMS, vs non-BMS patients were seen with respect to nearly all baseline demographic and clinical characteristics assessed with the exception of family history of MS (Table 2). Less than 1 % of the total cohort who met Criteria I criteria for benign status had progressive disease (SP, PP or SP). Removing these patients from the analysis made no difference in statistical significance of results, nor did considering them non-BMS due to their status as progressive patients (Table *2*). Positive predictors of conservative-criteria BMS at baseline included female sex (*p* < 0.0001) and younger age at onset (*p* < 0.0001). Patients with progressive-onset type of MS and those of African-American race were less likely to have BMS at baseline (Table 2). Of the 382 African-American patients included in the study cohort, 11.8 % were classified as having Criterion I BMS. In LR models, comparing benign (EDSS ≤2) with non-BMS patients with DD ≥10 years for both groups, findings were similar to those of univariate 2-group comparisons, except that BMS patients were more likely to have a later age at onset than non-BMS patients after adjusting for other variables in the model.

### Characteristics of criterion I benign patients at follow-up

Follow-up data were available for significantly fewer patients (*n =* 3583, 57.3 %) compared with the original cohort (median cohort follow-up 3 years, range 1–8 years). Of the 1237 (19.8 %) patients originally classified as having BMS at baseline per Criterion I, 788 (63.7 %) had follow-up data available (median follow-up 4 years, range 1–8 years). Of these patients, 64.8 % (*n =* 511) continued to present with BMS at follow-up, whereas the remaining 35.2 % of patients (*n =* 277) had progressed to non-BMS.

### Factors associated with loss to follow-up

Logistic regression analyses also were used to evaluate factors predicting loss to follow-up. The full model tested included sex, race, disease course, DD, age at disease onset, and Criterion I classification (i.e. benign vs non-BMS). Variables that predicted higher loss to follow-up included having SPMS (*p <* 0.0001) or PPMS (*p <* 0.05) course; longer DD (*p <* 0.05); and being in the non-BMS group (*p <* 0.0001).

### DMTs in benign patients by criterion I cohort at baseline and follow-up

Most patients on DMT at enrollment and/or follow-up were on 1 of 3 DMTs: (1) IFN-β1a (Avonex)—enrollment only, *n =* 33; follow-up only, *n =* 157; (2) IFN-β1b (Betaseron)—enrollment only, *n =* 27; follow-up only, *n =* 37; or (3) glatiramer acetate (Copaxone)—enrollment only, *n =* 9; follow-up only, *n =* 53. Eight patients were taking methotrexate alone at enrollment, and 1 at follow-up. One patient was taking subcutaneous IFN-β1a (Rebif) at enrollment, and 6 at follow-up. Other DMTs taken by ≤1.2 % of patients at enrollment or follow-up were cyclophosphamide, mitoxantrone (Novantrone), intravenous immunoglobulin, oral myelin, linomide, and azathioprine.

Two-hundred ninety-two patients were on DMT at both enrollment and follow-up, including 173 on IFN-β1a (Avonex), 32 on glatiramer acetate (Copaxone), 44 on IFN-β1b (Betaseron), and 1 on IFN-β1a (Rebif). The remainder of patients on DMT at both time points (*n =* 42) changed DMT between enrollment and follow-up. One hundred nine patients (14.7 %) had no history of DMT use at enrollment and did not use DMT during the study.

### Effect of DMT on continued benign status in criterion I cohort

Cox models were used to simultaneously evaluate significant baseline predictors of benign status and DMT use in *n =* 742 Criterion I baseline benign patients. The only significant predictors of remaining benign at follow-up in the final fitted model were Ever used DMT (*p* = 0.009) and longer DD (*p* = 0.007), both of which were protective (Table 3, Fig. 1a). Longer DD and DMT use were also the only variables remaining in the final model for the analysis in which DMTs were grouped into 4 categories. In this latter analysis, likelihood of remaining benign at follow-up for those who took DMT at time of enrollment but stopped taking DMT before follow-up was not significantly different from Never used DMT. However, a protective effect was seen for those patients taking DMT at follow-up (HR = 0.80; *p* = 0.037). Furthermore, the protective effect was stronger and more significant for patients taking DMT at both enrollment and follow-up (HR = 0.71; *p* = 0.006) (Table 3 and Fig. 1b).

## Discussion

Although various definitions for the classification have been proposed, BMS is generally characterized by the lack of disease progression and minimal disability 10 years or more after disease onset [2, 19, 22]. The considerable variation in the estimated frequency of BMS has the potential to influence treatment decisions because, historically, some clinicians were reluctant to prescribe DMTs for patients with little evidence of physical disability [10]. Our own data supports this observation. At time of enrollment into the study cohort, only 40.8 % of benign MS patients were on DMTs, whereas 81.8 % of non-BMS patients (more than twice the rate of BMS patients, *p <* 0.0001) were on DMTs (Table 2). Current recommendations propose DMTs for patients with MS from date of diagnosis without waiting to determine whether they meet the criteria for benign disease [11, 13, 23].

This large study, which included 6258 patients enrolled in the NYSMSC registry, evaluated the frequency and characteristics of BMS in a prevalence cohort including patients from 18 centers across NYS accrued over a 6-year time period. Patients receiving investigational DMTs were included in the study to maintain the completeness of the dataset. The large sample size of this prevalence cohort allowed us to identify the prevalence of BMS and compare demographic and clinical characteristics of BMS according to the 3 most commonly used classification criteria [2, 3, 18], as well as to identify prognostic factors associated with the disease course of BMS. Based on the classification criteria used, the overall percentage of patients with BMS at enrollment was 19.8 % to 33.3 % at baseline, which is in accordance with previous studies [2, 10].

Our data suggest that patients with SPMS, PRMS, and PPMS disease course are less likely to remain benign over time than those with RRMS disease course, which is consistent with results previously reported in the literature [10, 18, 24]. Both baseline and follow-up data support the recommendations of others that the term “benign” MS should be reserved for patients with RRMS disease course [2, 3, 7, 8, 18, 22]. In cross-sectional baseline analyses, several demographic, clinical, and treatment factors increased the likelihood of being classified as benign. However, in exploratory Cox model analyses of benign patients over time, use of DMT and longer DD were the only significant prognostic factors for continued BMS. The data surrounding the use of DMTs suggest that there are benefits of early preventive treatment even in the absence of signs of physical disability. However, while follow-up analyses were limited to patients with baseline EDSS ≤2.0 after 10 or more years of disease duration, correlation between EDSS score and DMT use was not directly studied herein. Nevertheless, these novel findings support the consensus statements issued by the NMSS in 2008 and the Consortium of Multiple sclerosis Centers (CMSC) in 2014 regarding early initiation of DMTs upon definitive MS diagnosis for patients and continuing treatment in those who are appropriate candidates for these agents [11, 12]. Use of DMTs early in the MS disease course has been supported by pharmacoeconomics studies that indicate early vs. delayed treatment with IFN-β, in particular, may be more cost-effective in the long term, as reduction of relapses, hospitalizations, and indirect costs, and gains in quality of life, and appear to outweigh the costs of DMTs [14, 15, 25].

Our findings should be considered also in context of the study limitations. The diagnosis of MS at baseline and at follow-up was not based on McDonald but on Poser criteria [21, 26, 27]. Length of follow-up included in the follow-up analyses, was not the same for each patient but varied from 1 to 8 years. With the exception of 2 patients who were exposed to DMTs in clinical trials during the follow-up period, study patients were primarily treated with first-generation DMTs. Furthermore, this was a historical study, and available data did not allow us to examine every potential prognostic factor for BMS, including MRI. For example, because data on specific symptom at onset were not available for the cohort studied (i.e. optic neuritis, sensory symptoms, and longer time intervals from onset to the second attack), some factors previously shown to be prognostic of BMS were not examined herein [10, 18, 24]. Another limitation is the missing data on cognitive status, as EDSS used within this period did not include the cognitive functional score. Similarly, additional factors may be prognostic for patient loss to follow-up, and we may not have identified all possible causes of missing follow-up data. Also, while this was an exploratory study and we evaluated factors that predicted loss to follow-up, it is still possible that bias may have been introduced due to loss to follow-up. Our finding that less than 1 % of progressive patients in the study cohort met criteria for BMS at baseline supports the notion that that progressive patients should not be classified as benign, regardless of whether they meet EDSS and DD criteria for benign classification.

Nevertheless, this study has several strengths, including the large, NYS-based, centralized dataset of patients with MS that includes almost 400 African-American patients, some of whom had BMS; a comparison of the prevalence of BMS according to 3 different definitions of benign status; and the availability of treatment data that allowed us to explore the potential effect of DMT on likelihood of continued benign status.

The results in the African-American subset of patients in our cohort are consistent with previous findings from our group and others that African-American patients with MS are likely to experience more rapid progression of disease than Caucasians [20, 28, 29]. However, while they were less likely to be classified as benign at baseline, African-American patients with BMS were just as likely to remain benign over time as Caucasians after controlling for other significant predictors of maintaining benign status. It is unlikely that this finding is the result of differential loss to follow-up or differing follow-up times because African-American race was not associated with loss to follow-up, and median follow-up years for African-Americans with BMS was not significantly different from that of other BMS patients (4.5 vs 4.0 years).

On the basis of our historical cohort study findings and those from various studies reported in the literature, we support the recommendation of Pittock and others to narrow the definition of BMS to an EDSS ≤2 after a minimum of 10 years’ DD, at least until more reliable predictors of BMS (i.e. genetic, biologic, or imaging biomarkers) have been fully developed and validated [2, 8, 22]. Many patients retrospectively classified as having BMS may ultimately transition to a non-BMS, progressive form of MS, even using these more conservative criteria [4, 5, 16, 19, 30].

## Conclusions

Taken in context with previously reported benefits of the early initiation of DMT for patients diagnosed with MS [4, 9, 16, 25], our findings suggest that early initiation and continued treatment with DMT may increase the likelihood of maintaining BMS classification over the course of patients’ lives, thereby ameliorating disability progression, and are worth further consideration and evaluation.

## Abbreviations

BMS, Benign MS; CDMS, Clinically definite multiple sclerosis; CMSC, Consortium of Multiple sclerosis Centers; DD, Disease duration; DMTs, Disease modifying therapies; EDSS, Expanded Disability Status Scale; FDA, Food and Drug Administration; HRs, Hazard ratios; IFN-β, Interferon beta-1; IRBs, Institutional Review Boards; LR, Logistic regression; MS, Multiple sclerosis; NMSS, National Multiple Sclerosis Society; NYS, New York State; NYSMSC, New York State MS Consortium; PPMS, Primary-progressive multiple sclerosis; PRMS, Progressive-relapsing multiple sclerosis; RRMS, Relapsing-remitting multiple sclerosis; SPMS, Secondary-progressive multiple sclerosis; SPSS, Statistical Package for Social Sciences; UDSMR, Centralized data management center

## Additional file

Additional file 1: Figure S1. Study cohort diagram. Data from New York State Multiple Sclerosis Consortium. (TIF 377 kb)
