# Supplementary figures and images for: Factors associated with benign multiple sclerosis in the New York State MS Consortium (NYSMSC)
Source: BMC Neurol. 2016 Jul 15;16:102. doi: 10.1186/s12883-016-0623-2 (PMC4946222; doi:10.1186/s12883-016-0623-2)

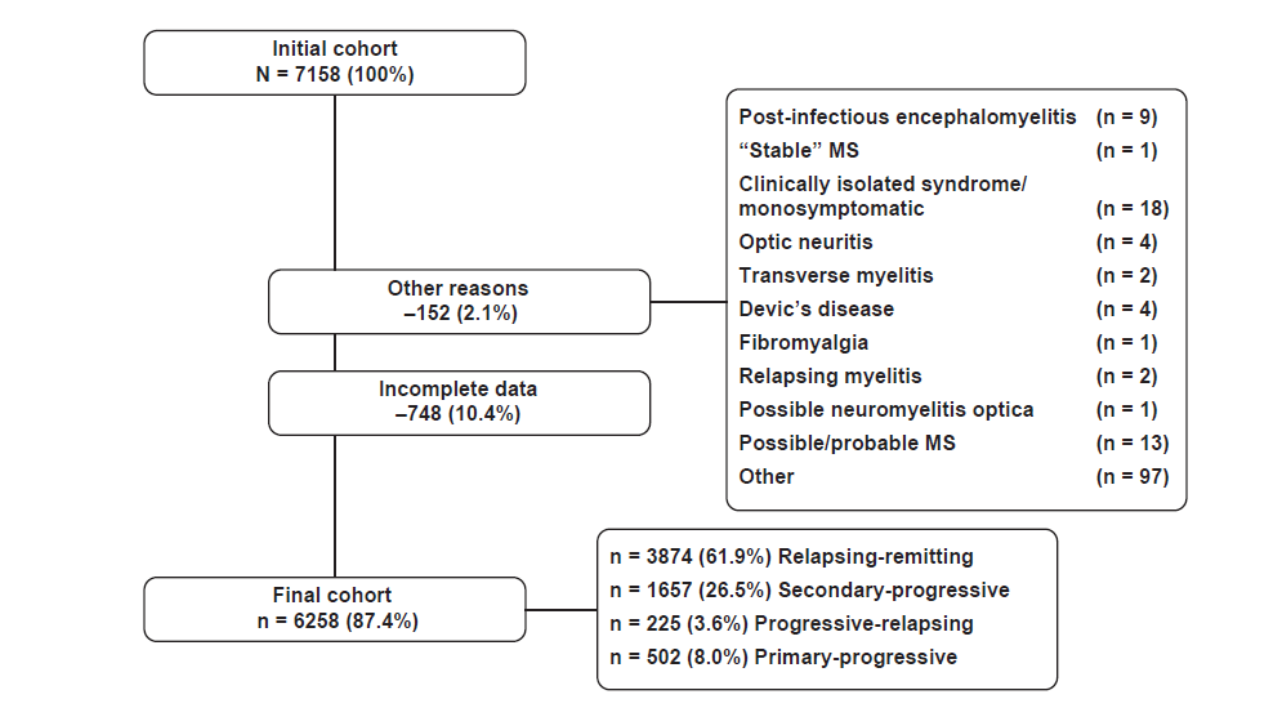

Supplement: Additional file 1: Figure S1. — Study cohort diagram. Data from New York State Multiple Sclerosis Consortium. (TIF 377 kb) [file 12883_2016_623_MOESM1_ESM.tif]
